# Exploring motor speech patterns in adults with minimally verbal autism spectrum disorder through surface electromyography

**DOI:** 10.3389/fnhum.2026.1757743

**Published:** 2026-07-30

**Authors:** Nishat F. Protyasha, Serena Pei, James R. Williamson, Laura Sarnie, Lisa Nowinski, Nataliya Kosmyna, Meredith Pecukonis, Paige Hickey Townsend, Sophia Yuditskaya, Christopher J. McDougle, Thomas F. Quatieri, Pattie Maes, Maria Mody

**Affiliations:** 1 Media Lab, Massachusetts Institute of Technology, Cambridge, MA, United States; 2 Lincoln Laboratory, Massachusetts Institute of Technology, Lexington, MA, United States; 3 Lurie Center for Autism, Massachusetts General Hospital, Lexington, MA, United States; 4 Harvard Medical School, Boston, MA, United States; 5 Athinoula A. Martinos Center for Biomedical Imaging Massachusetts General Hospital, Charlestown, MA, United States

**Keywords:** autism spectrum disorder (ASD), speech, feature extraction, machine learning, sEMG, signal processing

## Abstract

**Introduction:**

It is well known that standardized neuropsychological testing frequently fails to capture the true capacity and full range of abilities in individuals with minimally verbal autism spectrum disorder (mvASD) due to difficulties with motor speech skills. Here we used Surface Electromyography (sEMG), a non-invasive method that captures action potentials during muscle movements, to examine the motor basis of speech production challenges in adults with minimally verbal autism spectrum disorder (mvASD).

**Method:**

sEMG data were collected from 8 sensors placed on the face and neck while participants performed four speech tasks: imitation, naming and reading words, and a syllable repetition task (diadochokinetic, DDK). We compared adults with mvASD and neurotypical controls (NT) on RMS amplitude and mean correlation between the sEMG signals and movement complexity measures derived from auto and cross-correlation structures of the signal to capture the dynamic coordination of muscle activity during speech production. Principal component analysis was applied to reduce the eigenvalue features to a compact speech-motor representation used as input to leave-one-out cross-validated predictive models of group.

**Results:**

Across all speech tasks, the mvASD group consistently demonstrated stronger correlations between sensor signals from muscles on the face and neck compared to NT. The finding suggests tighter coupling of muscle activity reflecting potentially less differentiated muscle movement control during speech production. This is in keeping with a pattern of lower complexity of motor coordination related to reduced degrees of freedom in mvASD compared to NT participants.

**Conclusion:**

The findings extend previous work by demonstrating that sEMG features capture differences in muscle coordination patterns between adults with mvASD and neurotypical individuals across multiple speech tasks. Additionally, dimensionality-reduced EMG representations derived from imitation and diadochokinetic (DDK) tasks showed the strongest ability to distinguish between groups, suggesting that speech tasks that involve reproducing the utterances presented may be particularly sensitive to atypical speech-motor control in mvASD. These results support the potential of sEMG as an objective tool for characterizing speech-motor performance and informing assessment approaches for minimally verbal individuals.

## Introduction

Autism Spectrum Disorder (ASD) is a heterogeneous neurodevelopmental condition characterized by difficulties in social communication and the presence of restricted and repetitive behaviors. Although not a core diagnostic feature, motor issues are also commonly observed early on in development in ASD (Begum Ali et al., 2020; Bhat et al., 2022; Harris, 2017; Wilson et al., 2018). Recent epidemiological data further underscores this relationship, suggesting that motor milestone delays may affect more than 70% of the autistic population, highlighting the potential importance of motor impairments as a commonly associated feature of ASD (Pokoski et al., 2025). Among individuals with ASD, the minimally verbal subpopulation (mvASD) is characterized by a severe expressive language impairment, where an individual fails to develop functional spoken language beyond a limited repertoire of 30 words or less (Bal et al., 2016; Pecukonis et al., 2025).

To understand the verbal challenges in mvASD, it is important to distinguish between language and motor speech constraints. Language refers to the cognitive and symbolic system used to communicate. It is made up of receptive and expressive domains (Owens, 2020). Receptive language is the ability to comprehend vocabulary, grammatical structures, and social cues. Expressive language is the ability to output thoughts, ideas, and feelings through words, gestures, or writing. While these involve the mental selection of linguistic units, motor speech is the physiological process of translating the mental representations into perceptuomotor units. Disruptions to the neurophysiological mechanisms involved in this translation process can give rise to motor speech disorders such as apraxia of speech and dysarthria involving specific disruptions in speech subsystems (Duffy, 2013). These contrast with and are distinct from cognitive-linguistic disruptions underlying expressive and receptive language.

Standardized neuropsychological assessments used in clinical practice, however, may not fully capture the abilities in individuals with mvASD due to the motor speech challenges in this populations. These assessments often rely heavily on following verbal instructions, limiting their utility in mvASD and potentially underestimating true cognitive or communicative potential (Kasari et al., 2013). Moreover, because development in mvASD does not follow typical trajectories (Slušná et al., 2021), and given that these individuals frequently undergo intervention, alternative tools are needed to track changes with development or in response to intervention.

Surface electromyography (sEMG), a noninvasive method that captures action potentials during muscle movements, presents one potential tool for addressing this gap. It allows for the examination of orofacial muscle activity during speech production in individuals with mvASD. However, because sEMG involves the placement of surface electrodes and adhesive sensors, individuals with sensory sensitivities (common in ASD) may experience some discomfort, underscoring the importance of developing tolerable and adaptable recording setups. This technique is well-established in speech recognition research, where it has demonstrated high efficacy in detecting critical oromotor movements, despite the challenges associated with it (Betts et al., 2006; Stepp, 2012; Voltech et al., 2021). As such, sEMG may have potential applications in clinical measurement and assessment.

Chenausky et al. (2021) explored how motor speech abilities correlate with expressive language skills in individuals with ASD and found that motor speech irregularities may contribute to reduced speech intelligibility and limited expressive language in some individuals. Building on this, recent auditory-perceptual analyses have further classified these challenges as distinct motor speech irregularities, highlighting significant impairments in coordination and consistency (Maffei et al., 2024). Both motor speech issues and language impairments can constrain opportunities for effective communication, motivating further investigation into how such sensorimotor differences shape communicative development. In fact, numerous studies have shown that early motor difficulties have been associated with differences in language development (Bhat et al., 2022; Chenausky et al., 2021; Mody et al., 2017; Pecukonis et al., 2025). Interestingly, Pecukonis et al. (2025) found that fine motor skills predict expressive language not receptive language abilities in adults with mvASD. This is consistent with recent findings by Simarro Gonzalez et al. (2024), who demonstrated that fine motor proficiency significantly differentiates individuals across the verbal communication spectrum, further suggesting that motoric constraints may play an important role in expressive communication differences. Together, these studies argue for a more integrated approach in assessing language abilities, one that includes motor speech skills as a contributing factor, in children and adults across the autism spectrum (Croft et al., 2025; Mody and McDougle, 2019).

In summary, these results offer insights into potentially unique linguistic profiles of mvASD which are often overlooked in traditional standardized testing. They align with the work of Kasari et al. (2013), who discussed the need for alternative approaches to assessing minimally verbal individuals with mvASD. Our work aims to contribute to the growing body of literature examining the distinct ways in which individuals with mvASD engage with oral language through improved characterization of motor speech patterns, which may have implications for how assessments and interventions are designed. Specifically, we draw on sEMG to characterize the motor speech patterns in mvASD based on the notion of speech gestures (Browman and Goldstein, 1989). This research aligns with the trend towards integration of technology-based diagnostics in autism, moving the field toward more precise and accessible clinical characterizations (Mao and Zhu, 2026).

Building on prior work demonstrating group differences in speech-related muscle activity (Zulfikar et al., 2024), the current study explores whether surface EMG can quantify the complexity of orofacial motor coordination in mvASD across speech tasks with varying cognitive demands. The study presents an exploratory analysis of sEMG in mvASD compared to NT through a systematic series of experiments, drawing on insights into motor speech ability from our earlier work in this population.

## Methods

### Participants

25 mvASD (16 males, 9 females; age: 25.8 years ± 4.9) and 26 NT adults (13 male, 13 female; age: 23.9 years ± 3.5) participated in this study. They were part of a larger investigation (34 mvASD (22 male, 12 female) and 29 NT adults (14 male, 15 female) characterizing communication abilities in mvASD adults (Pecukonis et al., 2025). Participants with mvASD were recruited through multiple sources: the Lurie Center for Autism at Massachusetts General Hospital database, clinician referrals, local clinics, primary care practices, autism organizations, educational programs, and community outreach through online advertisements, social media, and printed materials. ASD diagnosis was confirmed by a clinical psychologist using the Diagnostic and Statistical Manual of Mental Disorders, Fifth Edition (DSM-5; American Psychiatric Association, 2022) symptom checklist and the Social Communication Questionnaire (SCQ; Rutter et al., 2003). The presence of a comorbid psychiatric disorder that was the primary focus of treatment (e.g., schizophrenia, bipolar disorder, severe major depressive disorder, severe anxiety disorder, substance use disorder), a known genetic disorder associated with ASD (e.g., Fragile X Syndrome, Tuberous Sclerosis), neurological problems (e.g., cerebral palsy, lifelong encephalopathy, in utero stroke, severe anoxia at birth, meningitis), voice pathologies (e.g., polyp or node on vocal folds), were in the exclusionary criteria. Minimally verbal (mv) status was based on expressive language skills that fell at or below the 1st percentile according to scores on the Expressive Vocabulary Test (EVT-3), in combination with parent report (Pecukonis et al., 2025).

NT adults were drawn from the local community through university and hospital recruitment websites, study announcements, and flyers posted in public areas. They did not meet criteria for ASD based on the clinician rated DSM-5 symptom checklist and did not have a family history of ASD in any first-degree family members.

All participants had normal or corrected-to-normal vision and hearing, and English as the primary language used at home. NT participants provided written informed consent. For mvASD participants, legal guardians or authorized representatives provided consent, with additional verbal/written assent obtained when possible. Procedures were approved by the Human Subjects Committee of the Mass General Brigham Institutional Review Board (protocol P000526).

During the screening visit, participants completed a battery of standardized assessments measuring cognitive, motor, language, and social abilities. Cognitive ability was assessed using the Stanford-Binet Intelligence Scales (SB-5)-Abbreviated Battery (Roid, 2003), motor dexterity and coordination with the Grooved Pegboard Test–dominant hand (GPT; Mathews and Klove, 1964) and Beery-Buktenica Developmental Test of Visual Motor Integration (VMI-6; Beery et al., 2010) and language skills with the Peabody Picture Vocabulary Test (PPVT-5; Dunn, 2018) for receptive vocabulary and the Expressive Vocabulary Test (EVT-3; Williams, 2018) for expressive vocabulary. Social communication and autism-related traits were measured using the Social Responsiveness Scale (SRS-2; Constantino, 2012), and attention/hyperactivity symptoms were assessed with the ADHD-Rating Scale: Hyperactivity-Impulsivity subscale (ADHD-HI) See Table 1 for complete details about the participants.

**Table 1:** Standardized test scores (means and standard deviations) for mvASD and NT groups.

Measure	mvASD (*n* = 25)	NT (*n* = 26)
	Mean (SD)	Mean (SD)
SB-5 NVIQ (raw)	14.64 (6.99)	28.54 (2.47)
SB-5 NVIQ (scaled)	2.48 (3.06)	10.46 (2.08)
PPVT-5 (raw)	128.23 (27.97)	220.04 (10.04)
PPVT-5 (standard)	50.36 (9.99)	108.48 (12.8)
EVT-3 (raw)	69.79 (31.9)	168.28 (11.04)
EVT (standard)	52.92 (9.6)	109.56 (10.72)
VMI-6 (raw)	14.4 (5.15)	27.54 (2.14)
VMI-6 (standard)	49.0 (10.9)	94.92 (11.55)
GPT (standard)	51.88 (15.41)	92.88 (20.25)
SRS awareness (t-score)	68.53 (8.64)	46.00 (7.10)
SRS social cognition (t-score)	71.56 (8.01)	45.28 (6.70)
SRS: communication (t-Score)	72.04 (9.49)	45.58 (8.16)
SRS: social motivation (t-score)	65.68 (11.97)	50.54 (9.58)
SRS RRB (t-score)	70.62 (10.19)	47.00 (8.92)
SRS total (t-score)	72.24 (9.41)	46.65 (7.89)
ADHD-HI	6.83 (4.35)	2.92 (5.33)

Standard scores between 85–115 and Scaled scores between 8–12 are within the average range, except for the SRS where higher *T*-Scores reflect greater symptom severity (</= 59: within normal limits). Raw scores were used in the analyses due to floor effects with standard scores.

Here we expanded on our previous work (Zulfikar et al., 2024), which demonstrated significant group differences in speech-related muscle activity between mvASD and NT. The study involved 26 participants: 12 mvASD (8 male, 4 female) and 14 NT (8 male, 6 female) between the ages of 19–32 years (23.85 years ± 3.99), which was a subset of the participants in the current study.

### Data collection protocol

Following the standardized testing visit, qualified participants returned for the sEMG session during which data was collected on a series of speech production tasks using a recording set up partially based on AlterEgo; a wearable system from our previous work, which measures EMG signals from the face, jaw, and neck with electrodes and was used in developing BCI devices (Kapur et al., 2018). Audio recordings were collected as part of a larger multimodal study examining speech, eye movements, and fine motor skills. Speech was recorded using a Sennheiser MKE 600 shotgun microphone (Sennheiser, Germany), which was connected to a Microsoft Surface Book 2 (Microsoft Corp., Redmond, WA) via a Rubix 44 audio interface (Roland Corporation, Japan). Audio signals were sampled at 48,000 Hz.

The tasks involved the verbal production of syllables sequences (DDK; diadochokinetic task) and target words, which included six core words: “book,” “dog,” “girl.” “apple,” “kitten,” and “potato” that were common to both subject groups. The words were selected based on language acquisition literature, to cover a range of phonetic features and syllable lengths and drawn from the participants’ repertoire and hence were familiar to all participants.

The same set of core words was used under three different speech production conditions: imitation, naming, and reading, representing increasing cognitive demand. Each word was attempted three times under each condition for a total of 54 words. All data collection was done at the Lurie Center by clinical research staff under the guidance of expert clinicians. The tasks were conducted in the presence of an experimenter to ensure procedural fidelity, while all vocalizations were recorded directly via laptop. We acknowledge that while the experimenter’s presence may introduce a social anxiety component, this standardized approach was maintained across all participants to ensure data quality.

*Imitation task*: Participants were asked to repeat the target words spoken by the experimenter one word at a time.

*Naming task*: Participants were asked to name the pictures of the target words presented one at a time on a laptop.

*Reading task*: Participants were asked to read aloud the target words presented one at a time on the laptop and point to the correct picture corresponding to each from a choice of two pictures below the target word on the screen.

*DDK* task: Participants were asked to repeat each of the syllables, /pa/, /ta, /ka/ (/pa-pa-pa/, /ta-ta-ta/, /ka-ka-ka/) as well as the syllables combined (/pa-ta-ka/,/pa-ta-ka, /pa-ta-ka/) in separate sequences, as many times as possible, each (i.e., same syllables and combination syllables) within a 10 s interval. Three runs were collected per syllable repetition condition. This task provides insight into both foundational speech motor skills as well as higher-order sequencing abilities involved in running speech as well as multisyllabic word production. The EMG data reveals the underlying muscle activity patterns during speech attempts.

Stimulus presentation and temporal synchronization were managed via Psychtoolbox on a dedicated laptop interface (Brainard, 1997). The audio data was collected using a custom-built MATLAB graphical user interface. Furthermore, video data provided a critical quality-control measure, allowing for the retrospective verification of electrode topography and the identification of non-speech artifacts that could confound the electromyographic profiles.

### Surface electromyography (sEMG)

**Figure 1:**
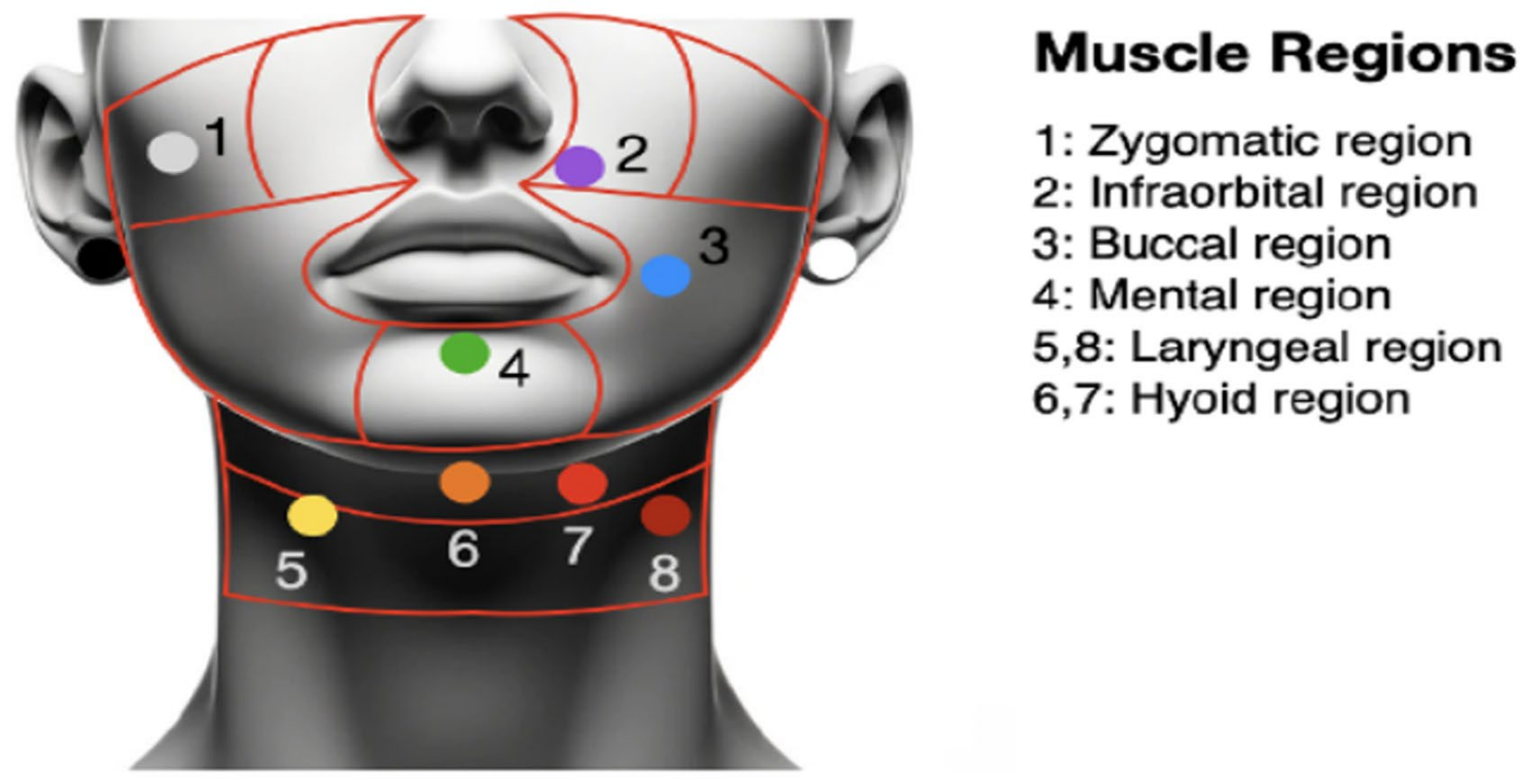
An eight-channel wearable surface electromyography (sEMG) system. Electrodes were placed on facial and neck muscles with reference electrodes on the ears. Adapted from Protyasha (2024).

An 8-channel wearable surface electromyography (sEMG) system was used to capture neuromuscular activity during speech (Figure 1). Based on validated research (Kapur et al., 2018; Kapur et al., 2020), Ag/AgCl electrodes were strategically placed on facial and neck muscles—specifically the orbicularis oris, zygomaticus major, and masseter—with reference electrodes on the ears to ensure signal stability.

**Figure 2:**
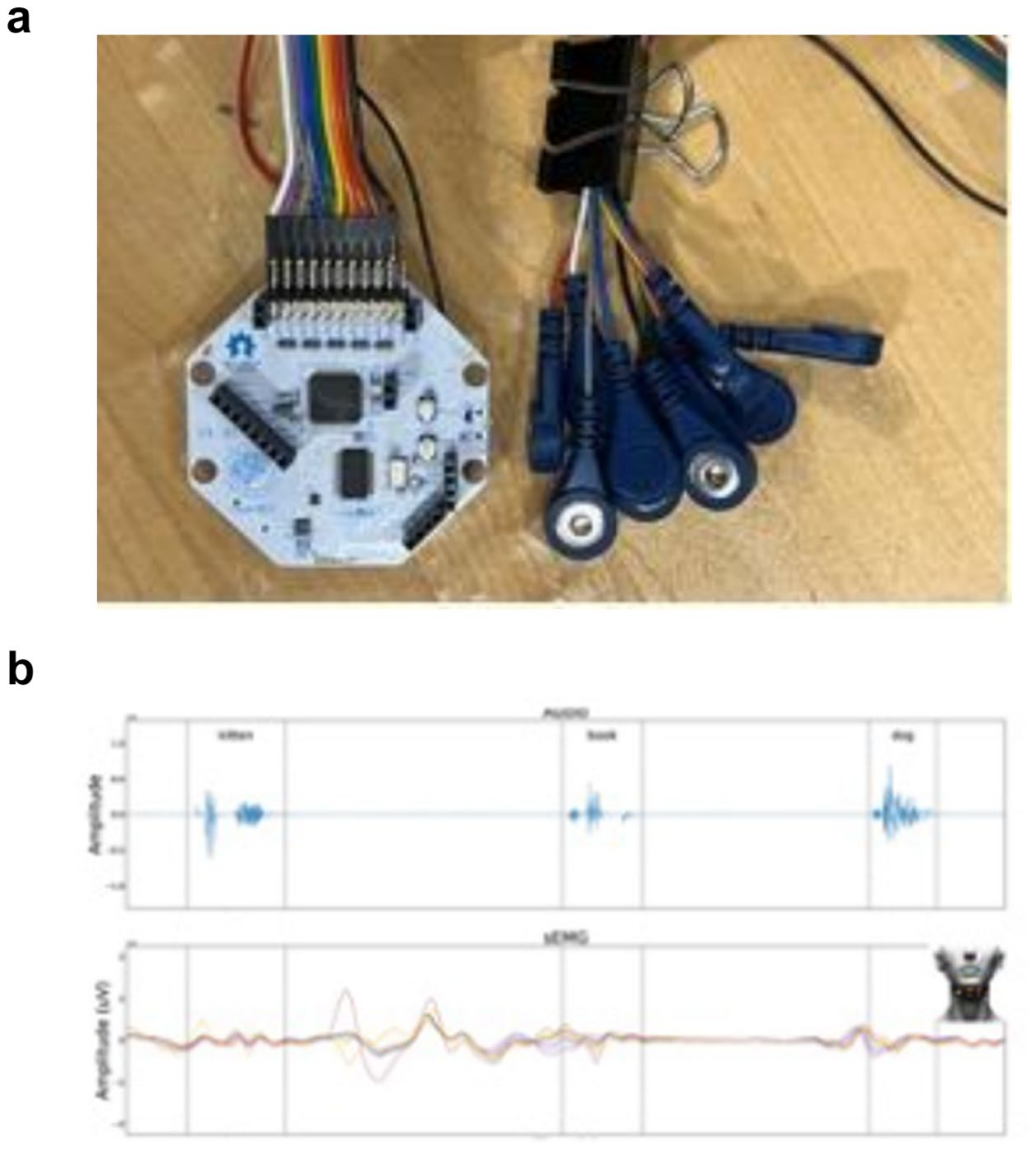
**(a)** OpenBCI Cyton biosensing board used for EMG data acquisition. This setup was used to capture orofacial muscle activity during speech production tasks. (Adapted from Protyasha, 2024). **(b)** temporally aligned auditory and sEMG signals corresponding to sample speech output.

Data acquisition was handled by the OpenBCI Cyton biosensing board (Figure 2). Operating at a 250 Hz sampling rate with a 24-bit analog-to-digital converter, the system recorded high-fidelity electrical disruptions in facial regions. We observed the data in real time using the Open BCI GUI. This included basic checks such as applying a bandpass filter to assess whether the signal looked noisy, or if an electrode had good contact. The real time processing had no impact on the data used in the analysis and was only used to ensure the raw data quality during acquisition.

### Treatment of data

In the current study, we used a larger sample to examine group differences in EMG. We followed a similar preprocessing and feature extraction pipeline used in our earlier study. Below we describe the analysis procedure in detail.

### Data preprocessing

Data preprocessing and analysis were conducted in Python using pandas, NumPy, SciPy, and scikit-learn. Visualization was performed using matplotlib and seaborn. Audio processing and feature extraction used librosa, soundfile, PyWavelets, and parselmouth. Praat software (Boersma and Weenink, 2018) was used for annotation.

First the speech data was annotated by trained annotators using Praat to mark onset and offset of each word utterance and DDK sequence. Then the sEMG data was aligned with the audio and segmented to correspond to the word utterances and DDK sequences (Figure 2). The raw sEMG signals for each utterance were corrected for DC offset and baseline drift using a high-pass filter, and power line noise was removed with iterative 60 Hz notch filters. Transient artifacts were removed via a Ricker wavelet transform, followed by a band-pass filter (0.5–8 Hz) to isolate relevant muscle activity to ensure signal quality and extract only the relevant muscle activity for further analysis. The study utilized a 0.5–8 Hz band-pass filter to specifically isolate the articulatory envelope. This lower frequency range is optimized to capture the temporal dynamics of ‘speech gestures’ and syllabic-rate coordination, which were the primary focus of our complexity and correlation analyses. This pipeline eliminates common noise sources while preserving physiologically meaningful signals. The audio and sEMG data were collected using different laptops (Microsoft Surface and Mac Air), so timestamps were not aligned across them. To align the timestamps, we took the difference between the calibration beep signal from the two recordings and applied this time delta to all utterances annotated from the audio. This ensured that our sEMG signal data was temporally consistent with the utterances from the audio recordings.

### sEMG features

*Power*: To estimate the signal power for each sEMG channel, the root mean square (RMS) was calculated. RMS values were average values across channels for each utterance within each task, which preserved the task level structure. We focused on the task level differences to average over a large enough sample size, while retaining the task level granularity to separate analyses for each task (naming, reading, imitation, DDK). We then calculated and compared the mean RMS for each word and DDK sequence between groups. Significance testing was performed using the glmmTMB package in R, employing a random mixed-effects model with a Gaussian distribution. The predictors for the mixed-effects models included group (ASD vs. NT) and type of task. This model accounted for inherent variability by treating participants and words/sequences as random effects. No group × task interaction term was included, and models were fitted separately for each task (naming, reading, imitation, DDK) and each word/sequence within that task. A group × task interaction was not applicable as the sole fixed effect in each model was group. The formula using glmmTMB with a beta family was: correlation ~ 1 + if_asd + (1 | subject) where if_asd is a binary indicator (ASD = 1, control = 0) and (1 | subject) allows each participant’s baseline correlation to vary. Models were fitted separately within each task and within each word/sequence.

*Correlation*: Pearson correlation coefficients, assuming zero phase delay (to explore how the eight channels activated instantaneously), were calculated between the sEMG channels to generate 8 × 8 correlation matrices. The mean values from these matrices were averaged per word for group-level analysis of global inter-muscle interactions. Averaging correlation values across all channel pairs yields a single scalar that indexes the overall level of inter-muscle coupling, which was motivated by our goal of characterizing global coordination at the level of a speech task. To compare the mvASD and NT groups, we utilized a mixed-effects model similar to the power analysis; however, a beta regression model was substituted for the Gaussian model to accommodate the bounded nature of correlation coefficients. The use of zero-lag correlations reflects our focus on the degree to which muscles are co-activated within the same analysis window rather than the temporal ordering of muscle recruitment. In the context of speech motor impairment, zero-lag correlations provide a stable, interpretable measure of the synchrony less sensitive to window-length choices and does not require assumptions about directional coupling. It is important to note that the averaging collapses muscle-specific information and the zero-lag correlation makes the measure insensitive to sequential coordination patterns. To prepare the data for beta regression, which requires dependent variables to lie within the (0, 1) interval, we used the absolute value of the Pearson correlation coefficients (|r|). This transformation allows the model to capture the overall magnitude of inter-muscle coupling, regardless of whether the phase relationship was positive or negative.

**Figure 3:**
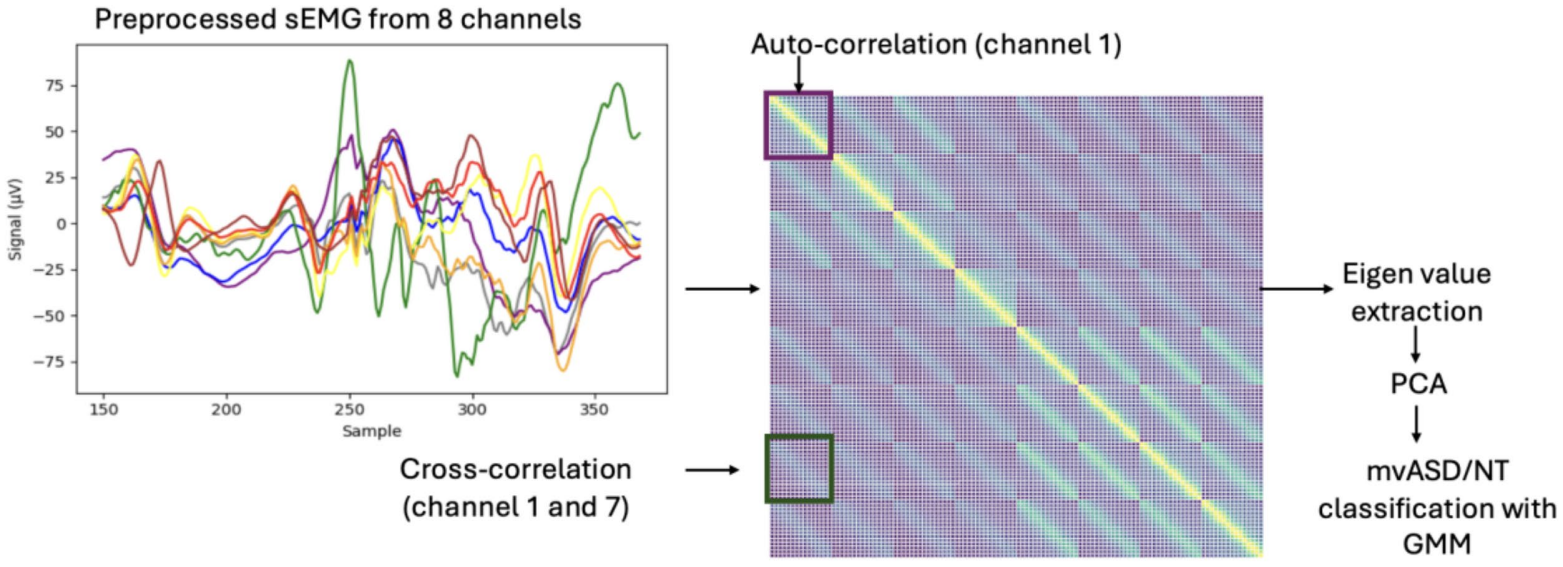
Visualization of autocorrelation across channels for feature extraction.

*Correlation structure*: We assessed muscle coordination using multivariate auto and cross-correlations across the eight sEMG channels (Figure 3). By applying time-delay embeddings (using 15 delays of 10 ms each), we expanded the signal dimensionality into 120 × 120 matrices that represent coupling strengths at various relative delays. The eigenvalues of these matrices were then rank ordered. Coordination complexity was inferred from the eigenspectra: a steep decline in eigenvalues suggests lower complexity (fewer degrees of freedom), whereas a more uniform distribution of eigenvalues indicates higher coordination complexity. Effect size was also calculated for these rank-ordered eigen spectra. These eigenvalue-based features were subsequently utilized to classify the mvASD and NT groups and to develop potential predictive models for clinical assessment scores.

To summarize, RMS served as a foundational index of overall muscle activation and motor effort, where elevated values indicate increased physical demand or relative inefficiency in executing a speech movement. We used pairwise correlations to measure inter-muscle coactivation and coupling in order to analyze the relationships between muscle groups; higher correlations suggest more synchronous, dependent activation patterns that effectively reduce the degrees of freedom available to the system. Finally, eigenvalue-based features were used to characterize the dimensionality and underlying structure of these correlation patterns, with a more concentrated eigenvalue spectrum reflecting lower coordination complexity and a more constrained rigid state of motor control. Based on our recent findings of acoustic differences in the speech patterns of individuals with mvASD (Quatieri et al., 2026), we draw on the above measures as part of an exploratory examination of the motor speech differences underlying the verbal challenges in mvASD.

Our approach builds upon established signal processing methods previously applied to audio-based features in studies of ASD (Talkar et al., 2025). Notably, similar eigenvalue-based analyses of correlation matrices have demonstrated reduced motor coordination complexity in children with ASD (Talkar et al., 2025) and in mvASD adults (Zulfikar et al., 2024), compared to NT; validating this methodology for characterizing speech-related muscle movements in clinical populations. The current application extends these principles to sEMG data as a means for quantifying orofacial motor control in mvASD individuals through physiological signals.

## Results

### sEMG features: mean RMS and correlations

The following analysis compares the statistical patterns of mean RMS and sEMG channel correlations between the mvASD and NT groups. The results in Table 2 reveal distinct differences in EMG activity between the two groups across the speech tasks. Compared to NT, the mvASD group showed significantly higher RMS amplitude only during the Imitation task. In contrast, correlation between sEMG signals across all channels was consistently stronger in the mvASD group for all tasks. Only the Imitation task yielded a consistent difference between groups on both sEMG features, showing significantly higher RMS amplitude and signal correlation between EMG sensors in mvASD compared to NT.

**Table 2:** (top) Mean amplitude (RMS) and mean inter-sensor signal correlation across words and syllables in each task, and *p*-values of the difference between mvASD and NT from fitting generalized linear mixed models.

Condition	mvASD mean (SE)	NT mean (SE)	*p*-value
RMS amplitude (μV)			
Naming	126.20 (16.66)	146.76 (31.89)	0.911683
Reading	122.48 (17.97)	139.31 (18.72)	0.075359
Imitation	137.53 (14.69)	86.31 (6.32)	0.000306*
DDK	101.85 (12.19)	125.97 (33.30)	0.482199

Condition	mvASD mean (SE)	NT mean (SE)	*p*-value
Correlation (r)			
Naming	0.57 (0.02)	0.50 (0.01)	0.0*
Reading	0.63 (0.01)	0.50 (0.01)	0.000156*
Imitation	0.56 (0.01)	0.52 (0.01)	0.000133*
DDK	0.55 (0.01)	0.48 (0.01)	0.0*

SE, Standard error.

*indicates significant *p*-values.

**Figure 4:**
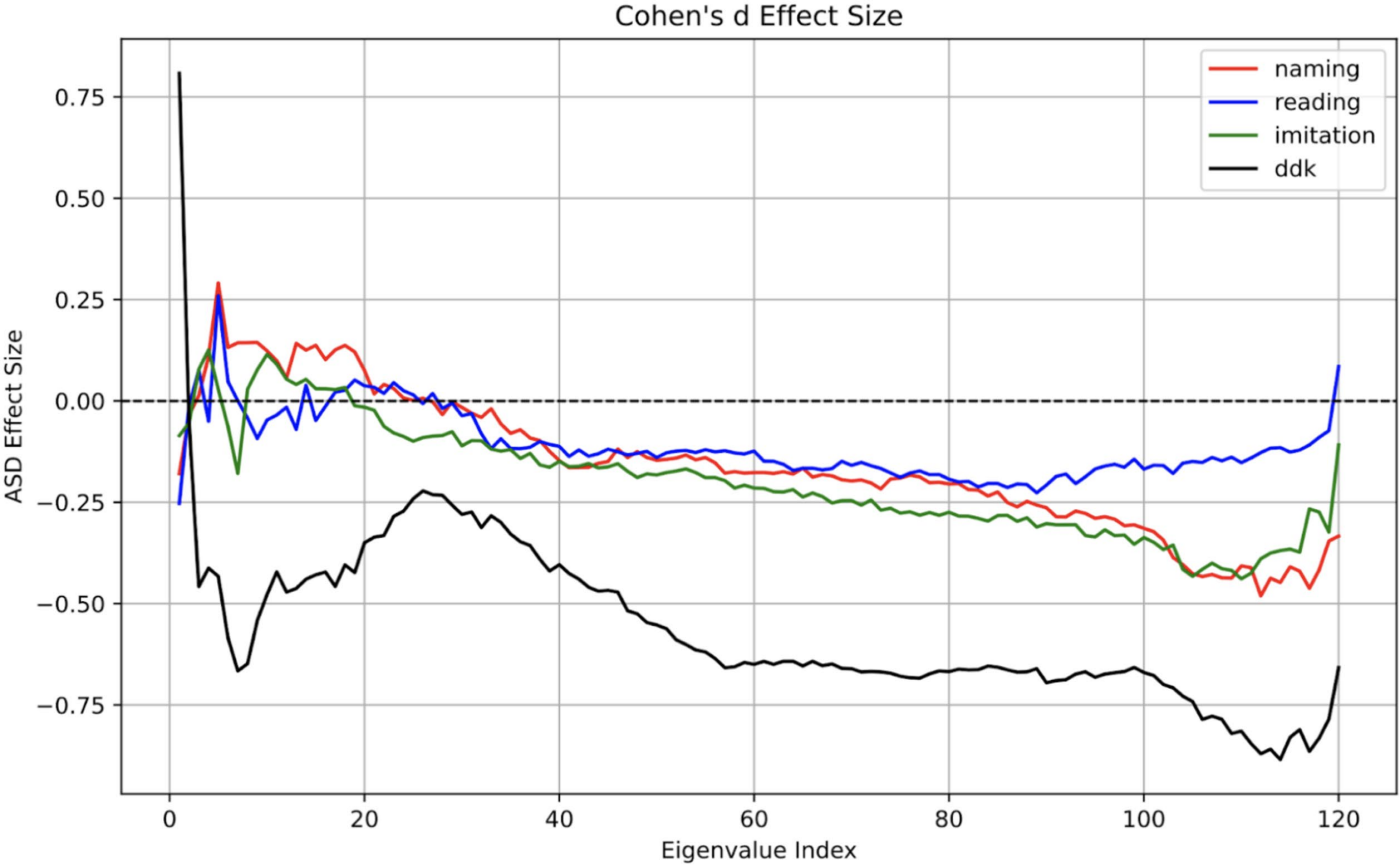
Cohen’s d effect size of EMG signals for naming, reading, imitation, and DDK tasks.

Across all speech tasks (imitation, naming, reading, and DDK), the mvASD group demonstrated stronger correlations between sensor signals from muscles on the face and neck compared to NT (see Table 2 and Figure 4). The finding suggests tighter coupling of muscle activity reflecting potentially less differentiated control of muscle movements during speech production in keeping with reduced complexity of motor coordination in mvASD than NT participants. In contrast, the mvASD group showed significantly higher RMS amplitude compared to NT only during the Imitation task.

### Complexity of motor movements

Figure 4 presents effect sizes of the difference between mvASD and NT across 120 eigenvalues, revealing distinct EMG activation differences between the groups across various speech tasks. The plot shows DDK with a steeper negative slope than the other tasks. Lower-index eigenvalues (leading components) represent dominant, low-dimensional coordination patterns that explain the majority of shared variance across muscles. These components are more likely to reflect task-specific coordination strategies and functional organization of muscle activity. In contrast, higher-index eigenvalues capture lower-variance, higher-dimensional structure, which may reflect more subtle or distributed coordination patterns that are less task-dependent and potentially more stable across conditions. For lower-index eigenvalues, the tasks exhibited smaller magnitude values, suggesting that mvASD and NT groups were relatively comparable for task specific coordination strategies. In the higher-index eigenvalues, the DDK task effect size reached nearly −1.0 around index 115, indicating that subtle, distributed coordination was where the mvASD group exhibited notable differences from the NT group. The overall pattern of differences suggests that simpler cognitive processes show smaller mvASD-related differences at lower-dimensional representations, while higher-dimensional sEMG patterns are consistently altered across tasks, especially in the DDK task.

### Classification

We implemented a logistic regression model to classify participants into mvASD and NT groups using PCA features derived from EMG recordings, with the full feature set consisting of 120 eigenvalue-based components.

For each speech task (DDK, Imitation, Naming, Reading), we averaged absolute autocorrelation eigenvalues across repetitions of each target word, producing a 120-dimensional feature vector per participant per word. We used 5-fold stratified cross-validation with per-word z-score standardization and PCA to retain components that explain 95% of training variance. The per-word principal component scores were concatenated to form the input feature vector to the logistic regression model (L2 penalty, max_iter = 1,000, random_state = 42). Performance was summarized as the mean AUC ± standard deviation across the 5 folds. EMG patterns associated with Imitation and DDK performance appeared to be the most effective in classifying mvASD and NT as seen in Table 3.

**Table 3:** Classification performance by task (AUC scores).

Task	Mean AUC (SD) (logistic regression)
DDK	0.7667 (0.2312)
Imitation	0.8167 (0.0697)
Naming	0.6667 (0.2485)
Reading	0.4000 (0.3201)

## Discussion

In this study, we used surface EMG to explore motor speech patterns in adults with mvASD compared with neurotypical controls across a variety of speech tasks (DDK, and imitation, naming and reading of single words). The tasks ranged from producing a string of syllables to a set of words familiar to the subjects and varied in the cognitive-linguistic demands on speech production.

EMG signal features showed the greatest discriminative potential for differentiating mvASD and NT participants during the imitation and DDK tasks, which required participants to repeat the target speech utterances. This pattern suggests that speech-motor differences in mvASD may be most apparent when participants are required to reproduce these speech targets. In contrast, naming and reading tasks produced weaker classification performance, indicating that the sensitivity of sEMG-based measures may depend on the speech demands imposed by the task.

Prior work suggests that differences in imitation in autism are not fully understood and may involve multiple domains, including attention, social motivation, motor coordination and action representation (Kilroy et al., 2020; McWeeny et al., 2025). As such, increased demands on these processes during imitation may have amplified group differences, reflected in the EMG signal. To the extent that the mvASD and NT groups differed across multiple domains, the observed EMG differences may be considered as phenotypic differences characteristic of ASD. We speculate that the demands that speech imitation makes on motor control and coordination, to reproduce the targeted utterances may be contributing to the differences in the mvASD. Based on complexity and logistic regression analyses, tasks like imitation and DDK hence may be well suited to capture motor profiles in mvASD.

In summary, the elevated amplitudes and coupling patterns we observed may be associated with the broader ASD phenotype, reflecting constrained coordination in the form of increased effort or inherent differences in task engagement. However, this result aligns with our prior work suggesting reduced degrees of freedom in speech motor coordination in this population (Quatieri et al., 2026; Zulfikar et al., 2024). It may well be that the increased amplitude reflects greater overall motor effort, whereas heightened coupling suggests a more constrained coordination strategy in which muscles are recruited in a less independent and more globally synchronized manner. Rather than selectively activating task-relevant articulators, the motor system may operate with fewer flexible control parameters, resulting in a tighter linkage among muscles. This pattern may potentially represent a compensatory strategy to stabilize speech output, or it may reflect fundamental differences in speech motor network organization that contribute to the production challenges observed in mvASD, particularly under tasks with higher perceptuomotor processing demands.

While RMS values provide a summary measure of overall muscle activation intensity, they do not capture the relational structure among muscles or the temporal dynamics of coordination. In the context of reduced degrees of freedom, the defining feature of mvASD motor behavior may not be how strongly muscles activate, but how they co-activate. Correlation-based features directly quantify patterns of inter-muscle coupling and coordination, preserving information about shared variability and synchronized control. By contrast, RMS collapses complex spatiotemporal dynamics into a single amplitude metric, potentially obscuring the constrained coordination patterns that differentiate groups. This likely explains why correlation features outperformed gross RMS amplitude in classification, as they are more sensitive to the organization of the motor system rather than its overall activation level. Exploratory analyses of behavioral test-score prediction in mvASD using the extracted EMG features were left out as they did not yield significant effects and were not central to the primary objective of characterizing group-level differences.

Several limitations should be considered when interpreting these results. First, the modest group size may limit power to detect subtle effects. However, adults with mvASD are critically understudied and difficult to recruit, and the magnitude and consistency of the motor-speech differences suggest that the findings are unlikely to be artifacts of sampling (Tager-Flusberg and Kasari, 2013). Our primary objective in this study was to examine inter-group differences between mvASD and NT participants. We considered clustering approaches using SRS severity scores but were restricted by the modest sample size and heterogeneity within the ASD group; further sub-clustering would substantially reduce statistical power and limit interpretability. Phenotyping, though, remains an important future direction requiring larger samples. To validate these findings, future studies should incorporate larger and more diverse samples. A more systematic comparison of mvASD and NT individuals across tasks that parametrically vary phonological complexity, motor demands, and cognitive load would help disentangle motor and cognitive contributions to speech production differences (Dromey and Benson, 2003; MacPherson, 2019; Saletta et al., 2018). Additionally, our study focused on adults with mvASD, and coordination patterns may differ across developmental stages. Finally, while EMG provides detailed insight into peripheral muscle activation, it does not directly measure neural mechanisms. Further work integrating neuroimaging methods will be necessary to clarify how altered neural control and cognitive-linguistic disruptions give rise to the observed motor speech coordination patterns. Nonetheless, sEMG enables investigation of speech as coordinated vocal tract gestures unfolding in articulatory space over time for linguistic signaling (Studdert-Kennedy, 1998; Iskarous, 2019).

In conclusion, by extracting features that quantify the structure and interdependence of muscle activation during speech tasks, we were able to classify mvASD with high accuracy specifically from the DDK and Imitation task. These findings contribute to a growing body of analysis exploring motor skills in mvASD and may be consistent with differences in motor coordination during speech production under tasks with varying perceptuomotor processing demands. These differences could reflect multiple underlying factors, but the present data do not allow us to distinguish between possible explanations. Pokoski et al. (2025) found an association between motor milestone delays and an earlier autism evaluation. Given that early identification of autism is a public health priority, this finding highlights the importance of assessing motor milestone delays for timely intervention and improved developmental outcomes. Future research should explore whether EMG-based metrics can track developmental changes and intervention outcomes on tasks including speech over time, potentially offering a more sensitive index of progress than traditional speech and language assessments. This biologically grounded approach may support more inclusive and precise methods for assessing and understanding communication in mvASD.

## Data Availability

The data will be made available through public access repositories on completion of the study. Requests to access the data should be directed to luriecenterresearch@mgb.org.
